# Genomic virulence features of *Beauveria bassiana* as a biocontrol agent for the mountain pine beetle population

**DOI:** 10.1186/s12864-023-09473-4

**Published:** 2023-07-10

**Authors:** Janet X. Li, Kleinberg X. Fernandez, Carol Ritland, Sharon Jancsik, Daniel B. Engelhardt, Lauren Coombe, René L. Warren, Marco J. van Belkum, Allan L. Carroll, John C. Vederas, Joerg Bohlmann, Inanc Birol

**Affiliations:** 1 Michael Smith Laboratories, University of British Columbia, 2185 East Mall, Vancouver, BC V6T 1Z4 Canada; 2 Canada’s Michael Smith Genome Sciences Centre, BC Cancer Agency, 570 W 7th Ave #100, Vancouver, BC V5Z 4S6 Canada; 3 Department of Chemistry, University of Alberta, 11227 Saskatchewan Drive NW, Edmonton, AB T6G 2G2 Canada; 4 Department of Forest and Conservation Sciences, University of British Columbia, Vancouver, BC V6T 1Z4 Canada; 5 Department of Botany, University of British Columbia, Vancouver, BC V6T 1Z4 Canada

**Keywords:** *Beauveria bassiana*, Forest health genomics, Pest control, Fungal metabolism

## Abstract

**Background:**

The mountain pine beetle, *Dendroctonus ponderosae*, is an irruptive bark beetle that causes extensive mortality to many pine species within the forests of western North America. Driven by climate change and wildfire suppression, a recent mountain pine beetle (MPB) outbreak has spread across more than 18 million hectares, including areas to the east of the Rocky Mountains that comprise populations and species of pines not previously affected. Despite its impacts, there are few tactics available to control MPB populations. *Beauveria bassiana* is an entomopathogenic fungus used as a biological agent in agriculture and forestry and has potential as a management tactic for the mountain pine beetle population. This work investigates the phenotypic and genomic variation between *B. bassiana* strains to identify optimal strains against a specific insect.

**Results:**

Using comparative genome and transcriptome analyses of eight *B. bassiana* isolates, we have identified the genetic basis of virulence, which includes oosporein production. Genes unique to the more virulent strains included functions in biosynthesis of mycotoxins, membrane transporters, and transcription factors. Significant differential expression of genes related to virulence, transmembrane transport, and stress response was identified between the different strains, as well as up to nine-fold upregulation of genes involved in the biosynthesis of oosporein. Differential correlation analysis revealed transcription factors that may be involved in regulating oosporein production.

**Conclusion:**

This study provides a foundation for the selection and/or engineering of the most effective strain of *B. bassiana* for the biological control of mountain pine beetle and other insect pests populations.

**Supplementary Information:**

The online version contains supplementary material available at 10.1186/s12864-023-09473-4.

## Background

The mountain pine beetle (MPB; *Dendroctonus ponderosae*) is an irruptive bark beetle that infests and kills most native pine (*Pinus*) species in its range [1]. It is prone to periodic outbreaks that can cause the mortality of trees over large areas. The most recent outbreak, beginning in the late 1990s, was exacerbated by a warming environment that enhanced beetle survival, and wildfire suppression that increased the abundance of susceptible host trees [2]. To date, MPB has affected approximately 18 million hectares of lodgepole pine (*P. contorta*) forests in western North America, with over 50% of mature lodgepole pine trees killed [3]. The cumulative economic loss to western Canada alone has been estimated at 90 billion CAD [4]. The extreme size of the outbreak, together with a climate change-induced increase in suitable habit, facilitated rapid range expansion into new regions including northern British Columbia and eastward over the Rocky Mountains into north-central Alberta [5, 6]. Eastward expansion has facilitated infestations within populations of lodgepole pine, jack pine (*P. banksiana*), and their hybrid (*P. contorta x P. banksiana*); hosts with limited defensive capacity due to a lack of coevolutionary interactions with MPB [7–10]. MPB poses an alarming continental threat, as jack pine is the predominant pine species that extends across the boreal forest to the Great Lakes and the Atlantic Coast of Canada [10–14]. Efforts to mitigate the spread of MPB have been hampered by the lack of effective control methods [15]. MPB escapes conventional pesticides as it spends all but a few days during dispersal flight beneath the bark of host trees [16]. Furthermore, mutualistic ophiostomatoid blue stain fungi introduced into trees by MPB limit the efficacy of systemic insecticides [17]. Hence, the physical removal of infested trees, through felling and burning or salvage harvesting, is currently the main mitigation tactic for MPB [15]. However, this approach is often costly, logistically difficult, and prone to failure. Therefore, development of a cost-effective and ecologically viable management approach to control MPB populations is critical to minimize its continued spread and impact.

*Beauveria bassiana* (Bb) is an entomopathogenic fungus that efficiently kills many species of insect pests. Different strains of this fungus are widely used in agriculture and are approved as safe biological insecticides that do not significantly hamper pollinating insects [18]. Bb kills various bark beetle species, although some strains have narrow host species spectra [19–21]. Some strains attack forest insects, including European spruce bark beetle (*Ips typographus*) [22], red palm weevil (*Rhynchophorus ferrugineus*) [23], pine shoot beetle (*Tomicus piniperda*) [24], and spruce beetle (*Dendroctonus rufipennis*) [21]. Although certain strains of Bb are lethal to MPB [25, 26] and other bark beetles in the laboratory [19, 21], field tests failed to demonstrate desirable mycosis propagation and control of beetle populations [21]. These field observations may be associated with ultraviolet (UV) damage of the Bb, lack of drought tolerance as well as inefficient conidial density and viability. Because of the extensive effort involved in testing Bb strains with insects, most studies focus on only a few strains. This limits the initial examination of strain virulence associated with biocontrol potential for MPB.

Due to the diverse phenotypes and numerous strains of Bb [27], genomic or molecular markers to determine the potential efficacy of the fungus as a biocontrol agent, potentially for MPB [25, 26], are highly desirable. In our previous study, we evaluated 93 Bb isolates from various culture collections worldwide and selected strains for their different phenotypes and virulence toward MPB [26]. Selected strains were characterized based on colony morphology, growth rate, infection rate, conidial capacity, and pigmentation and assessed for virulence against MPB in the laboratory. The strains were then categorized into three phenotypic groups. The pigment oosporein was of particular interest; oosporein is an extensively studied red dibenzoquinone virulence factor that promotes infection and may also afford UV resistance [28–31]. While not directly responsible for insect mortality, oosporein, a polyketide synthase (PKS) product, has been shown to contribute to immune system evasion by the fungus, and to suppress the insect host immune system to result in infection [29]. This compound has been shown to demonstrate antimicrobial and cytotoxic activities [31] and is widely studied in Bb. Group I strains produced high levels of oosporein and displayed the highest levels of virulence against MPB. Group II strains grew thin, cream-coloured colonies with no pigment and had the lowest virulence against MPB, requiring a higher conidial titer, and group III strains grew felty, yellowish colonies with intermediate virulence levels [26].

Here, eight promising isolates of *B. bassiana* across three phenotypic groups were used for genome sequencing and transcriptome analysis. These analyses identified genome and transcriptome signatures of each strain, particularly those associated with virulence, secondary metabolite biosynthesis and UV resistance. The presence and absence of secondary metabolite biosynthetic gene clusters (BGC) was assessed to identify those associated with virulence against MPB. BGC types include non-ribosomal peptide synthetases (NRPS), polyketide synthases (PKS) and terpenoids synthases. Of special interest was the oosporein BGC, a PKS cluster that was identified in all sequenced Bb strains and differentially expressed in some. Phylogenetic relationships, gene content and differential expression were also assessed, demonstrating large scale differences between the eight strains.

## Results

### Qualitative detection of oosporein production in *B. bassiana* strains

The eight Bb strains representing the three phenotypic groups are UAMH 299, UAMH 299-UVR and UAMH 1076 (Group I); UAMH 298, UAMH-298-UVR, UAMH 4510 and UAX-29 (Group II); and 110.25 (Group III) [24]. Strains UAMH 298-UVR and UAMH 299-UVR are UV resistant mutant derivatives of UAMH 298 and UAMH 299, respectively [24]. Oosporein detection was performed qualitatively using liquid chromatography-mass spectrometry (LC–MS) through tenfold serial dilutions over 5, 10 and 15 days of fungal growth in liquid culture. The limit of detection for oosporein was determined as 100 ng/mL. Oosporein was detected in the highly virulent group I strains UAMH 299, UAMH 299-UVR and UAMH 1076, as well as transiently in the group II strain UAMH 298-UVR (Table 1) after 5 days.

**Table 1:** Presence/absence of oosporein in purified mycelial supernatant extracts detected on LC–MS

**Strain/Day of detection**	UAMH 299	UAMH 299-UVR	UAMH 298	UAMH 298-UVR	UAMH 1076	UAX-29	UAMH 4510	110.25
Day 5	Yes	Yes	No	Yes	Yes	No	No	No
Day 10	Yes	No	No	No	Yes	No	No	No
Day 15	Yes	No	No	No	Yes	No	No	No

### Genome assembly of *B. bassiana s*trains

Table 2 provides a summary of the Supernova genome assemblies for the eight Bb isolates. The assemblies ranged from 33.95–40.04 Mb in length, slightly higher than the reported genome for the reference *B. bassiana* strain ARSEF 2860 (33.70 Mb) [32], but concordant with other assemblies for the species [33]. Assembly contiguity varied between the genomes; the most contiguous assembly (110.25) had an N50 length of nearly 1.19 Mb while the least contiguous assembly (UAX-29) had an N50 length of 115.66 kb. Despite the range in contiguity, all assemblies were highly complete in the gene space, containing 95.30–96.53% of the 4,494 Benchmarking Universal Single-Copy Ortholog (BUSCO) genes for the hypocreales_odb10 lineage in a single copy.

**Table 2:** Genome assembly metrics for the eight *B. bassiana* isolates under study

Strain	Group	n	Assembly Length (Mb)	Largest Contig (Mb)	N50 Length (kb)	GC content (%)	Repeat Length (Mb)	Complete Single-Copy BUSCOs (%)
UAMH 299	I	566	33.95	2.34	369.66	51.50	2.35	4,326 (96.26%)
UAMH 299-UVR	I	591	33.89	1.96	261.87	51.51	2.31	4,324 (96.22%)
UAMH 298	II	468	34.84	2.42	651.04	51.51	2.45	4,319 (96.11%)
UAMH 298-UVR	II	504	34.82	2.34	501.87	51.51	2.43	4,316 (96.04%)
UAMH 1076	I	797	35.26	0.91	152.23	51.49	2.61	4,317 (96.06%)
UAX-29	II	1,076	37.44	1.72	115.66	48.78	5.11	4,331 (96.37%)
UAMH 4510	II	503	40.04	2.66	649.37	47.24	8.01	4,283 (95.30%)
110.25	III	337	34.71	3.72	1,185.59	48.95	4.29	4,338 (96.53%)

### Phylogenetic inference

**Fig. 1:**
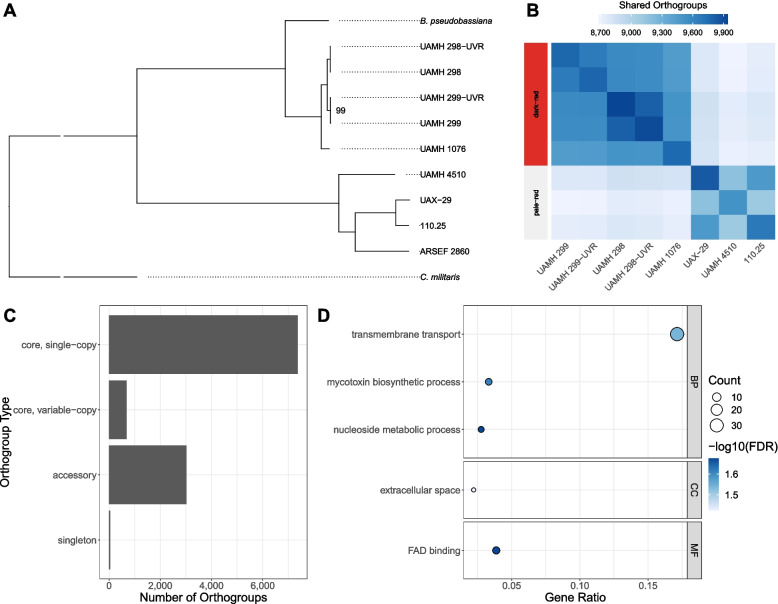
Comparative genomics analysis of *B. bassiana* isolates. **A** Best-scoring maximum-likelihood phylogenetic tree based on 3,939 complete, single-copy BUSCO proteins. The phylogeny includes the eight *B. bassiana* strains under study, reference strain ARSEF 2860 and *Beauveria pseudobassiana* strain KACC 47484. *Cordyceps militaris* strain CM01 included as the outgroup. All non-labelled nodes have bootstrap support values of 100. The tree is truncated to emphasize branching and tips. **B** Orthogroups shared between pairs of isolates. Dark-red and pale-red groups are highlighted on the left. **C** Summary of orthogroup types inferred among isolates. **D** GO enrichment analysis results for dark-red group specific orthogroups. Gene Ratio refers to the number of orthogroups that are annotated in a specific ontology (BP, CC or MF) term in proportion to all annotated dark-red group specific orthogroups

A phylogenetic tree (Fig. 1A, Fig. S1) was estimated using 3,939 complete, single-copy BUSCO genes shared between the eight Bb isolates under study, the reference strain ARSEF 2860, *Beauveria pseudobassiana,* and *Cordyceps militaris* as the outgroup. The eight Bb isolates fell into two distinct clusters with high bootstrap support. The group I strains UAMH 299, UAMH 299-UVR and UAMH 1076 clustered with the group II strains UAMH 298 and UAMH 298-UVR, while the other strains (UAX-29, UAMH 4510 and 110.25) formed a separate cluster with ARSEF 2860. Oosporein was detected in the UAMH 298-UVR strain, suggesting the capacity for oosporein production by both UAMH 298 and UAMH 298-UVR. Due to this result and the phylogenetic clustering of these two strains, they are now placed in group I. The two phylogenetic clusters were designated as the dark-red and pale-red groups, respectively, and the strains within these groups were designated as the dark-red and pale-red strains. The dark-red group clustered with *B. pseudobassiana* rather than ARSEF 2860, suggesting that they may belong in a different species.

### Genome annotation and identification of *B. bassiana* orthogroups

The eight genomes contained between 10,117 and 10,754 predicted genes. These predicted gene counts are comparable to the 10,366 genes encoded in ARSEF 2860 [30]. There was very little alternative splicing predicted, with the vast majority of genes having only a single isoform. Like the genome assemblies, the annotations were highly complete, containing 94.79–97.46% complete, single-copy BUSCOs. An orthogroup is a set of two or more genes descended from a single ancestral gene, analogous to an ortholog, but allowing for the comparison of several species or strains rather than a pair [34]. A total of 11,120 orthogroups (OGs) were inferred between the isolates, with each isolate containing between 9,514 and 9,936 OGs. A summary of the annotation and orthogroup results is presented in Table 3.

**Table 3:** Genome annotations and orthogroups

Strain	Genes	Transcripts	Complete, Single-Copy BUSCOs (%)	Orthogroups	Genes in orthogroups
UAMH 299	10,180	10,197	4,299 (95.66%)	9,776	10,176
UAMH 299-UVR	10,165	10,188	4,303 (95.75%)	9,779	10,162
UAMH 298	10,406	10,421	4,306 (95.82%)	9,936	10,400
UAMH 298-UVR	10,372	10,391	4,314 (95.99%)	9,909	10,371
UAMH 1076	10,457	10,472	4,295 (95.57%)	9,761	10,408
UAX-29	10,638	10,656	4,352 (96.84%)	9,839	10,543
UAMH 4510	10,754	10,777	4,260 (94.79%)	9,514	10,522
110.25	10,117	10,134	4,380 (97.46%)	9,674	10,072

There were 8,059 core orthogroups present in all eight isolates, and 7,370 of these were present in a single copy in each. There was a considerable amount of gene content variation between isolates, with 3,016 accessory OGs present in two or more but not all strains (Fig. 1C). Dark-red and pale-red strains shared more orthogroups with strains in the same group than with strains in the other (Fig. 1B). These group-specific overlaps represent a subset of accessory orthogroups as they are shared among two or more, but not all strains. The dark-red strains had 533 unique OGs and 461 were unique to the pale-red strains. While pale-red group specific OGs were not enriched for any Gene Ontology (GO) terms, five GO terms were significantly enriched in the dark-red group specific OGs (Fig. 1D), including mycotoxin biosynthetic process, transmembrane transport and extracellular space. The most commonly occurring protein domain, present in 18 dark-red group specific OGs, was the Major Facilitator Superfamily (MFS) domain. OGs involved in mycotoxin biosynthesis and pathogenesis included four heat-labile enterotoxin alpha chain domain-containing genes and six mycotoxin biosynthesis protein UstYa domain-containing genes.

### Biosynthetic gene cluster mining

The antiSMASH algorithm identified 39 to 49 biosynthetic gene clusters in each genome of the eight isolates. The dark-red strains contained more clusters than the pale-red strains, and the most commonly occurring cluster types among all strains were non-ribosomal peptide synthetase, type 1 polyketide synthase (T1PKS) and terpene synthase. Oosporein production was detected in the group I strains (UAMH 299, UAMH 299-UVR and UAMH 1076) and briefly in UAMH 298-UVR but not in the other strains, however the oosporein gene cluster (T1PKS) was identified in all genome assemblies (Dataset S2). The main biosynthetic genes responsible for oosporein synthesis (*OpS1*-*OpS7*) were present in all strains with 90.50–97.75% identity to Bb ARSEF 2860. The putative cell surface protein (*Ops9*) was not found in any of the eight strains, and the putative heat-labile enterotoxin IIB, A chain *(OpS10*) was absent in UAMH 298, UAMH 298-UVR, UAMH 1076 and UAMH 4510.

Additionally, several previously characterized gene clusters were found in all eight isolates. The cluster responsible for beauvericin synthesis (NRPS) was nearly complete, with all genes except the predicted pseudogene glycolate oxidase (*orf4*) identified in all strains. The dimethylcoprogen (NRPS) and clavaric acid (triterpene) clusters, both of which only contain one gene, were found in all strains, as well. Finally, the squalestatin S1 PKS cluster was identified in all strains, but only two of the five genes were detected in each.

The bikaverin PKS gene cluster was present in all dark-red strains, but absent in the pale-red strains, and the bassianolide NRPS gene cluster was identified in the pale-red strains, but not in the dark-red strains. UAMH 299 uniquely contained the trichodiene-11-one terpene cluster, and UAMH 1076 contained four unique clusters including sespendole (indole-terpene) and nivalenol (sesquiterpene). A summary of the antiSMASH results is provided in Dataset S1. Common, group- and strain-specific clusters identified by the KnownClusterBlast algorithm are listed in Table 4, and results from ClusterBlast are provided in Dataset S3.

**Table 4:** Common, group- and strain-specific gene clusters predicted by antiSMASH’s KnownClusterBlast algorithm

Strains	Accession	Name	Cluster Type
Common Clusters			
All	BGC0001249	dimethylcoprogen	NRPS
All	BGC0001720	oosporein	PKS
All	BGC0001248	clavaric acid	triterpene
All	BGC0001839	squalestatin S1	PKS
All	BGC0000313	beauvericin	NRPS
Pigment Group-Specific			
Dark-Red	BGC0000030	bikaverin	PKS
Pale-red	BGC0000312	bassianolide	NRPS
Strain-Specific			
UAMH 299	BGC0001811	trichodiene-11-one	terpene
UAMH 1076	BGC0001966	BII-rafflesfungin	NRPS
UAMH 1076	BGC0001882	chrysoxanthone A/chrysoxanthone B/ chrysoxanthone C	PKS
UAMH 1076	BGC0001278	nivalenol/deoxynivalenol/3-acetyldeoxynivalenol/15-acetyldeoxynivalenol/neosolaniol/calonectrin/apotrichodiol/isotrichotriol/15-decalonectrin/T-2 toxin/3-acetyl T-2 toxin/trichodiene	sesquiterpene
UAMH 1076	BGC0001775	sespendole	indole;terpene

The cluster names and types presented in the table above were determined and reported by the antiSMASH algorithm. See Supporting Information for the chemical structures of the gene cluster products

### Differential gene expression between dark-red and pale-red strains

**Fig. 2:**
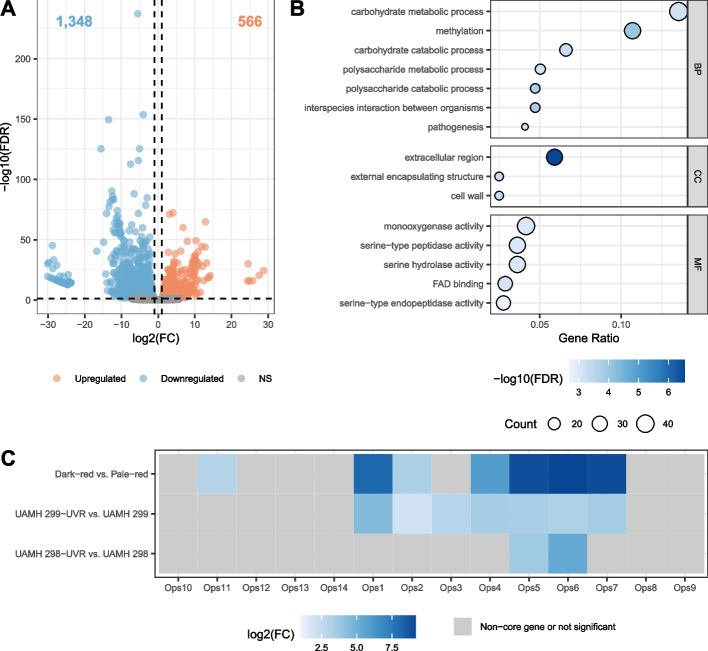
Summary of differentially expressed genes between dark-red and pale-red strains. **A** Volcano plot demonstrating number of upregulated, downregulated and not significant (NS) genes. **B** GO enrichment analysis results for differentially expressed genes. Top 15 most significant genes (as measured by FDR) are presented. Gene Ratio refers to the number of differentially expressed genes that are annotated in a specific ontology (BP: Biological Process, CC: Cellular Component or MF: Molecular Function) term in proportion to all expressed genes. **C** Differential expression of oosporein biosynthetic cluster genes. Genes on the X-axis are arranged in their genomic order within the cluster. FC: Fold change

Differential gene expression was identified between dark-red (UAMH 298, UAMH 298-UVR, UAMH 299, UAMH 299-UVR, and UAMH 1076) and pale-red (UAX-29, UAMH 4510, 110.25) strains. Bb strain ARSEF 2860 coding sequences (CDS) CDS [32] were used as the transcriptome reference. Of the 10,364 genes in Bb strain ARSEF 2860, 10,027 had an average normalized read count greater than 0, and there were no count outliers detected. 1,914 genes were differentially expressed, of which 566 were upregulated and 1,348 were downregulated in the dark-red strains compared to the pale-red strains (Fig. 2A). GO enrichment analysis identified functional patterns in the large set of differentially expressed genes. There were 27 enriched GO terms at FDR < 0.05, including interspecies interaction between organisms, pathogenesis and extracellular region, and the 15 most significantly enriched terms are presented in Fig. 2B.

There were several enriched terms related to metabolism and catabolism of small molecules, aromatic amino acids, carboxylic acids and carbohydrates. Fifteen lipase genes were significantly differentially expressed between the dark-red and pale-red strains, six of which were upregulated in the dark-red strains (Group I). Several chitinase, chitinase-like, putative endochitinase and chitosanase precursor enzymes were also differentially expressed. Additionally, four hydrophobin genes were significantly downregulated in the dark-red strains, including both *Hyd1* and *Hyd2*, and these genes were annotated with the GO terms cell wall and external encapsulating structure. Furthermore, 27 cytochrome P450 genes were differentially expressed in dark-red strains, annotated with monooxygenase and oxidoreductase activity. These differentially expressed genes are known virulence factors that are involved in Bb infection during conidial attachment and penetration of the insect cuticle [35].

The GO terms interspecies interaction between organisms and pathogenesis shared several overlapping genes, including two heat-labile enterotoxins whose expression levels were upregulated in dark-red strains. Several genes in the oosporein BGC were annotated with these terms, as well, and were highly upregulated in the dark-red strains (Fig. 2C).

### Differential expression and transcriptional regulation of oosporein BGC

All main biosynthetic genes in the oosporein cluster (*OpS1*-*OpS7*) were upregulated with the exception of *OpS3*. Many genes in the oosporein BGC displayed log_2_ fold change (LFC) values greater than 5, including *OpS1*, the core PKS enzyme responsible for synthesizing the precursor orsellinic acid [36]. *OpS3* is a Gal4-like Zn(2)-Cys(6) transcription factor (TF) and is required for the expression of the main biosynthetic genes *OpS1*-*OpS7*, including *OpS3* itself [36]. *OpS11*, a putative evolved D-lactonohydrolase [31], was also upregulated. *Bbsmr1* encodes a zinc finger TF and is a negative regulator upstream of *OpS3* but was not differentially expressed between the dark-red and pale-red strains. *Bbmsn2,* another negative regulator of oosporein production, was upregulated in the dark-red strains, contradictory to their phenotype and the differential expression results. Co-expression analysis identified 332 genes with expression levels differentially correlated to *OpS1* and *OpS3* between dark-red and pale-red strains, including several Zn(2)-Cys(6), b-ZIP and other transcription factors. These included genes that were both positively and negatively correlated with *OpS1* and *OpS3* expression, representing potential positive and negative regulators of oosporein gene expression and production. Most showed no correlation in pale-red strains, suggesting novel transcription factor activity or co-regulation in dark-red strains. Furthermore, the Velvet protein-encoding gene *VeA*, a master regulator of secondary metabolism [37], was differentially co-expressed with both genes, showing negative expression correlation in both dark-red and pale-red strains.

### Differential expression between UV resistant and wildtype strains

**Fig. 3:**
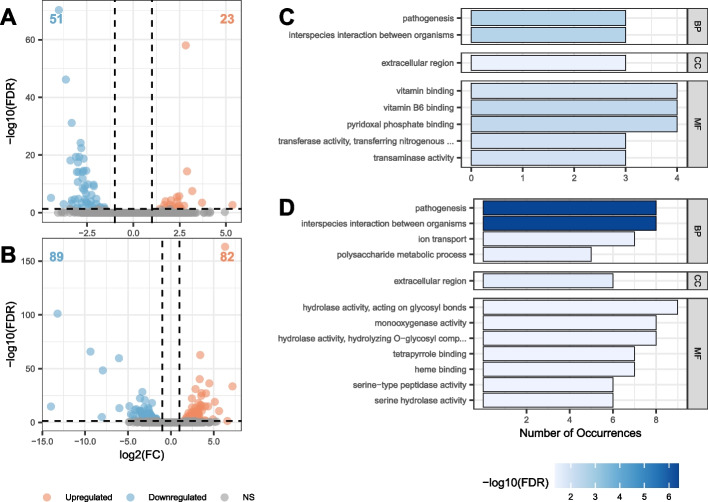
Summary of differential expression results for UVR strains and WT counterparts. **A** Volcano plot presenting differentially expressed genes between UAMH 298-UVR and UAMH 298. **B** Volcano plot presenting differentially expressed genes between UAMH 299-UVR and UAMH 299. **C** GO enrichment results for DE genes in UAMH 298-UVR. **D** GO enrichment results for DE genes in UAMH 299-UVR

Differential expression analysis identified 23 upregulated and 51 downregulated genes in UAMH 298-UVR (Fig. 3A) compared to the UAMH wildtype strain. The differentially expressed genes were significantly enriched for eight GO terms (Fig. 3C), including vitamin binding (FDR < 0.012), extracellular region (FDR < 0.039) and pathogenesis (FDR < 0.0029). Two genes encoding glucose repressible protein *Grg1* were downregulated in UAMH 298-UVR, and a gene with a heme-dependent catalase-like domain was upregulated. Two glutathione-S-transferase genes were upregulated, one of which was *OpS6* of the oosporein BGC. Additionally, the oosporein BGC gene laccase 2 (*OpS5*) was upregulated. In the UAMH 299-UVR strain, 82 genes were upregulated and 89 were downregulated compared to UAMH 299 (Fig. 3B). 12 GO terms were significantly enriched among these differentially expressed genes (Fig. 3D), including pathogenesis (FDR < 4.15e-7), monooxygenase activity (FDR < 0.046), tetrapyrrole binding (FDR < 0.046), and extracellular region (FDR < 0.026). Two multicopper oxidase genes were differentially expressed, including the laccase 2 gene (*OpS5*). The conidial pigment biosynthesis scytalone dehydratase *Arp1* was downregulated, and the DNA replication complex GINS subunit *Sld5* was upregulated. Additionally, a gene encoding a MAC1 interacting protein involved in stress response was downregulated in UAMH 299-UVR. Eight genes were differentially expressed in both UV resistant derivatives, including UDP-glucosyltransferase, laccase 2 and glutathione-s-transferase. These three genes were all upregulated in both strains.

## Discussion

Bb virulence is highly variable and displays an extremely broad host range [26, 38]; however, a lack of characteristic phenotypic variation within the species has made phylogenetic delineation difficult [39]. With the increasing accessibility of genome sequencing, phylogenomic techniques are being used to infer evolutionary relationships between strains [33, 39]. The genome assemblies and annotations generated for this work are highly complete in the gene space, providing confidence that they contain the necessary information for comparative analyses. Phylogenetic analysis of shared BUSCO sequences placed the eight Bb strains into two distinct groups. UAMH 298 and UAMH 298-UVR clustered with the group I strains and were placed in group I accordingly, and this cluster of strains was examined as a unit for increased virulence and oosporein production. Oosporein was transiently detected in UAMH 298-UVR and may have been produced in the UAMH 298 strain below the limit of detection during the collection times. Bb ARSEF 2860 has been shown to produce oosporein in previous studies as well [36], but our findings suggest an increased capacity for oosporein synthesis by the dark-red strains, as well as differential content of oosporein BGC genes. Interestingly, the dark-red strains formed a clade with *B. pseudobassiana* rather than the reference Bb strain. The *B. pseudobassiana* species was first described by Rehner et al. in 2011, using a multilocus phylogeny of 68 *Beauveria* strains based on the partial gene sequences of *Rpb1*, *Rpb2*, *Tef 1-a* and the nuclear intergenic region Bloc [39]. As the name suggests, *B. pseudobassiana* is phenotypically similar to *B. bassiana*, and has also been studied for its entomopathogenic properties [40, 41], but has slightly smaller conidia. Further investigation using whole genome annotations would be necessary to confirm the phylogenetic placement of the dark-red strains and *B. pseudobassiana;* however, these results show that the two isolate groups have undergone different evolutionary histories and demonstrate different virulence and oosporein production levels.

Orthogroup analysis revealed a large amount of gene content variation among strains, particularly when comparing the dark-red and pale-red groups. Accessory orthogroups, which may have arisen through gene duplication and neofunctionalization or were lost in an ancestral species, provide unique capabilities not necessary to survival but potentially confer an evolutionary advantage. OGs unique to the dark-red group were enriched for several GO terms including mycotoxin biosynthetic process and transmembrane transport, and therefore may contribute to increased toxin production and secretion, or differential expression of virulence-related genes in the dark-red strains. Of note, the dark-red strains contained several unique toxin-encoding genes, including four heat-labile enterotoxins and six genes containing the mycotoxin biosynthesis protein UstYa domain. Heat-labile enterotoxin is a compound produced by enterotoxigenic *Escherichia coli* that causes diarrhoeal diseases in animals. This toxin belongs to the same family as the cholera, pertussis and diphtheria toxins [42], and is hypothesized to provide an alternative mode of infection by compromising insect gut epithelium after ingestion of Bb spores [43]. In the phytopathogenic *Ustilaginoidea virens*, the *ustYa* gene is involved in the biosynthesis of a toxic cyclic tetrapeptide called ustiloxin A [44]. Homologues of this gene are present in various filamentous fungi, and while their purpose is still unknown, they have been shown to be involved in ribosomal peptide synthesis in several *Aspergilli* species [45] and could therefore be involved in secondary metabolism.

Biosynthetic gene cluster mining results further demonstrated the genomic variability between strains. More clusters were predicted in the dark-red strains than the pale-red strains, which could reflect an increased capacity for secondary metabolism and virulence. Notably, the BGC responsible for synthesizing the red pigment bikaverin in *Fusarium* species [46] was present only in the genomes of the dark-red strains. Bikaverin exhibits antibiotic and antifungal activity [47] and could function similarly to oosporein by outcompeting organisms in the host microbiome during infection. Additionally, the virulence factor bassianolide was unique to the pale-red strains and may contribute to the insecticidal activity in these strains. The differential content of biosynthetic gene clusters reveals that the genomic factors driving virulence are diverse and variable between Bb strains. Further characterization of these biosynthetic gene clusters and their products is necessary to determine their role in Bb virulence.

Several genes of interest were identified through differential expression analysis between the UVR strains and their wild type parents. Many were involved in stress response, monooxyenase activity and copper metabolism, all of which are crucial to protecting against UV radiation and oxidative stress. A gene encoding a MAC1 interacting protein was downregulated in UAMH 299-UVR. This gene contains a CFEM domain, which is a fungal-specific protein domain involved in a variety of biological functions including cell wall and cell membrane maintenance [48]. Early studies of MAC1 in *Saccharomyces cerevisiae* revealed that the protein is a transcription factor responsible for regulating genes required for copper metabolism and stress response [49], suggesting potential involvement in oxidative stress response. Two highly similar glucose-repressible protein encoding genes were downregulated in UAMH 298-UVR, one of which was annotated as *Grg1*. In *Podospora anserina*, transcription of *Grg1* was induced by carbon starvation and aging, and its expression was significantly lowered in a long-lived mutant with decreased cellular copper levels and oxidative stress. Downregulation of these glucose-repressible genes in UAMH 298-UVR could therefore be a result of decreased oxidative stress. Furthermore, upregulation of a heme-dependent catalase-like domain containing gene and two glutathione-s-transferase genes in UAMH 298-UVR could improve the strain’s ability to remove damaging reactive oxygen species (ROS). Testing individual variants using genetic engineering approaches such as CRISPR/Cas9 editing would help to determine which variants specifically confer UV resistance.

Given the phenotypic diversity between Bb strains, it was originally hypothesized that the group I strains contained the oosporein BGC while the other strains did not. The core biosynthetic genes *OpS1*-*OpS7* were identified in all eight strains by antiSMASH, suggesting that the high levels of oosporein production in the dark-red strains are a result of regulatory factors or differential expression. The transcriptome data corroborated this hypothesis, demonstrating high levels of upregulation of *OpS1*, *OpS2*, *OpS4*-*OpS7* and *OpS11* in the dark-red strains, but the absence of upregulation for *OpS3* raised additional questions about the regulation of this gene cluster. Surprisingly, some oosporein cluster genes were upregulated in the UVR strains as well. Since oosporein is a pigment, it could potentially play a role in absorbing UV radiation, thus protecting these strains from oxidative stress. Another pigment that is known to play a protective role in UV radiation is melanin. Some fungi use L-dopa as a starter molecule and a laccase enzyme for the biosynthesis of melanin [50], however, it remains to be seen whether the upregulated *OpS5* is involved in the production of melanin in the UVR strains of Bb.

Regulation of the oosporein BGC is not well understood, but some regulatory factors have been identified. *Bbmsn2*, a zinc finger TF and stress response protein, negatively regulates oosporein in a pH-dependent manner [51]. This protein is also required for fungal penetration through the insect cuticle [52]. Unfortunately, the genetic mechanisms underlying oosporein repression by Bbmsn2 have not been elucidated. Another zinc finger TF BbSmr1 was identified as an upstream regulator of the oosporein BGC, likely through regulation of the OpS3 transcription factor [31]. *BbSmr1* was not differentially expressed, and *Bbmsn2* was significantly upregulated in the dark-red strains. Since Bbmsn2 acts as a repressor for oosporein production, upregulation of this gene should cause a decrease in oosporein synthesis, therefore contradicting with the observed phenotype of the dark-red strains. Differential co-expression analysis identified several potential transcription factors involved in regulating the expression of *OpS1* and *OpS3*. Many of these were Zn(2)-Cys(6) type TFs, which is the same type of TF as OpS3. These TFs may be involved in regulating oosporein gene cluster expression and oosporein production upstream of *OpS3*. Furthermore, the Velvet protein-encoding gene *VeA* is of interest, as it is responsible for several processes including conidiation, secondary metabolism and stress response in Bb [37]. This protein may be involved in processes related to virulence in the dark-red strains. The differential co-expression results demonstrated a negative regulatory relationship between *VeA* and *OpS3*, contradicting the understanding of this protein’s function. This result is inconclusive; however, it is possible that there is an intermediate positive regulator of *OpS3* whose expression is negatively regulated by *VeA*, or vice versa. Given the fact that a complete oosporein BGC is present in the pale-red group, oosporein expression may be induced in these strains when infecting a host, and that such a condition is not replicated under laboratory growth conditions. The mechanism by which oosporein in the pale-red group might be upregulated remains to be elucidated.

## Conclusions

This work has characterized genomic and transcriptomic features of eight Bb isolates and how they contribute to increased virulence and oosporein production. New signatures of virulence, namely significant patterns in gene content and differential expression of genes related to pathogenesis, metabolism of small molecules and oxidoreductase activity were identified in the highly virulent dark-red strains. Oosporein biosynthetic cluster genes were upregulated in the dark-red group, and novel putative regulatory factors for the BGC were inferred through co-expression analysis. Additionally, several genes of interest involved in UV resistance were identified through differential expression analysis between UVR and wild type strains. This broad-scale analysis reveals major genetic patterns that contribute to the phenotypes of these isolates. While this work has identified genes and pathways of interest, biological function cannot be directly inferred from bioinformatic analysis alone. The results of these analyses will enable future functional validation in vivo. Our findings contribute greatly to the understanding of biological factors involved in the efficacy of *Beauveria bassiana* as a biological control agent. This work provides a foundation for future research and engineering of *B. bassiana* for the sustainable control of the mountain pine beetle and other insect pests related to agriculture, forestry and human health.

## Methods

### *B. bassiana* strains

The eight strains of Bb analyzed during this study were obtained from several culture collections previously [26]. The strains and their phenotypes are described in Table 1 of this study.

### Extraction and detection of oosporein from *Beauveria bassiana*

Eight morphologically similar Bb strains, with variable pigmentation, were selected and grown to obtain detection limits for oosporein production. The starting fungal inoculum was reactivated using Czapex-Dox Yeast Extract Agar medium, and incubated at 25 °C for 4–6 weeks, or until conidial lawn is at its maximum. The conidia were harvested from the agar media, titered, and inoculated a 25 mL Czapex-Dox Yeast Extract Broth (CDBYE) medium in a 250 mL Erlenmeyer flask to a final concentration of 1 × 10^7^ conidia/mL and incubated at 28 °C for 72–96 h at 175 rpm. After incubation, a 1 mL inoculum was used to inoculate a 250 mL CDBYE medium in a 1000 mL Erlenmeyer flask and incubated at 28 °C at 175 rpm. After 5 days of incubation, duplicate mycelial mixtures (2 × 30 mL) were harvested through centrifugation at 10,000 × *g* for 15 min at 4 °C. The supernatant was transferred to a new sterile conical tube and the resulting mycelial pellet was washed twice with ice-cold TE buffer (10 mM Tris/1 mM EDTA, pH 8.0), and the supernatant was combined with the previous sample. The mycelial cultures were incubated further, and the extraction process was carried out again after day 10 and 15. The supernatant was extracted with ethyl acetate (4 × 20 mL), concentrated *in vacuo*, and purified through high-performance liquid chromatography (HPLC). Oosporein concentration was detected using LC–MS through comparison to a previously synthesized standard [53]. Lastly, the limit of detection of oosporein was performed using tenfold serial dilutions and analyzed via LC–MS.

### DNA Sample preparation for genomic analysis

Bb strains were grown to establish high quality draft genome reference sequences. The starting fungal inoculum was obtained from frozen mycelial stocks and re-activated using Czapex-Dox Yeast Extract Agar (CDAYE) medium, and incubated at 25 °C for 4–6 weeks, or until the conidial lawn is at its maximum. The conidia were harvested from the agar media, titered, and inoculated a 100 mL CDBYE broth medium in a baffled 500 mL Erlenmeyer flask to a final concentration of 1 $\times$ 10^7^ conidia/mL and incubated at 28 °C for 72–96 h at 175 rpm. Mycelia were harvested through centrifugation at 5000 g$\times$ for 20 min at 4 °C. The resulting mycelial pellet was washed twice with ice-cold TE buffer, and flash frozen in liquid nitrogen, and stored at -80 °C until processing. Quality control analysis for bacterial contamination was performed for all samples. An aliquot of the mycelia was serially diluted ten-fold up to 1.0 $\times$ 10^6^ and all dilutions were spread-plated, in quadruplicate, on Standard Methods Agar (SMA, BD Difco Laboratories, Sparks, MD, USA) with 100 U/mL nystatin. Duplicate plates were incubated at either 28 °C or 35 °C for 28–72 h to assess the bacterial load. Frozen mycelial pellets were sent to the Michael Smith Laboratories, University of British Columbia, for DNA extraction.

### DNA Extraction

Fungal DNA was isolated from lyophilized mycelium (~ 5 g) with a modified protocol of Doyle and Dickson [54]. The modifications included adding 3% mercaptoethanol to the lysis extraction buffer, incubation at 60 °C for 45 min and washing the initial pellet with 75% ethanol with 10 mM ammonium acetate. An additional solvent cleaning with 1:1 phenol:chloroform-isoamyl solution was included after incubation with RNAse and proteinase K at 37 °C. The concentration and quality of DNA was verified with a Nanodrop 2000c (ThermoFisher Scientific, Waltham, MA, USA), Quantiflor (Promega Corporation, Madison, WI, USA) and 0.8% agarose gel. 3–4 µg total DNA for all 8 strains was sent to Canada’s Michael Smith Genome Sciences Centre for sequencing.

### Whole genome sequencing

A microfluidic partitioned library was created using the Chromium system (10 × Genomics, Pleasanton, CA, USA). Gel beads-in-Emulsion (GEMs) were produced by combining DNA, Master Mix, and partitioning oil in the 10 × Genomics Chromium Controller instrument with the microfluidic Genome Chip [PN-120216] (10 × Genomics). The DNA in each GEM underwent isothermic amplification as a barcode was added to each fragment. Barcoded fragments then underwent Illumina library construction, as per the Chromium Genome Reagent Kits Version 2 User Guide [PN-120229].

The resulting library was assessed for quality using the Agilent 2100 Bioanalyzer (Agilent Technologies, Santa Clara, CA, USA) and a DNA 1000 assay. The median insert size was 550 bp. The library was quantified using a Quant-iT dsDNA High Sensitivity Assay Kit on a Qubit fluorometer (Invitrogen, Waltham, MA, USA) prior to library pooling and size corrected final molar concentration calculation for Illumina HiSeqX sequencing with paired-end 150 base reads. The Long Ranger BASIC pipeline (v2.1.3) was run on the raw reads to perform read trimming, barcode error correction, whitelisting and barcode assignment [55]. The reads were downsampled by barcode to 18 million reads (approximately 80X sequencing depth) for further analyses.

### De novo genome assembly

The genomes were assembled using Supernova v2.1.1 [56], which leverages the long-range information provided by linked reads. Since read coverages were higher than the recommended range of 38-56X for assembly, 15 million reads were selected (approximately 56X coverage) using --maxreads = 15,000,000. The output FASTA files were created using the --style = pseudohap option which generated a single record per scaffold, and scaffolds shorter than 1 kb were excluded from the final assembly. The draft genomes were polished by aligning 80X downsampled reads to their respective genomes using BWA-MEM v0.7.17r1188 [57, 58] and supplying these alignments to Pilon v1.23. BUSCO v5.1.2 [59] was run to assess the genic completeness of the assemblies using the hypocreales_odb10 database in genome mode, and other assembly metrics were calculated with QUAST v5.0.2 [60]. EDTA v1.9.4 [61] and RepeatModeler v2.0.1 [62] were used to identify and annotate repetitive sequences within the polished assemblies. The coding sequences of Bb strain ARSEF 8028 [33] were supplied to EDTA to ensure that gene sequences were excluded from the resulting libraries. All identified repeats were merged with the RepBase [63] database of eukaryotic repeat sequences (v23.12), and redundant sequences were removed using the cleanup_nested.pl script from EDTA [61]. This custom repeat library was used as input for RepeatMasker v4.1.1 [64] to annotate and soft-mask repetitive sequences in the polished genome assemblies.

### Phylogenetic inference

Evolutionary relationships between the strains were inferred using RAxML v8.2.12 [65]. The Bb reference strain ARSEF 2860 [32] and *B. pseudobassiana* strain KACC 47484 (unpublished; GenBank accession: GCA_003267905.1) were also included in the analysis, as well as *C. militaris* strain CM01 [66], which was set as the outgroup with the -o option. Single-copy BUSCO sequences were used to generate the phylogeny as annotations were not available for the *B. pseudobassiana* genome assembly. Multiple sequence alignments were generated for the shared BUSCO sequences with MAFFT v7.475 [67], selecting the appropriate strategy automatically (--auto), and the resulting amino acid alignments were concatenated into a single matrix. The phylogeny was generated from a rapid Bootstrap analysis and search for the best-scoring Maximum Likelihood tree, using the PROTGAMMAAUTO model of amino acid substitution and 100 bootstrap replicates.

### Genome annotation and orthogroup inference

Genome annotation was performed using the MAKER (v2.31.10) pipeline [68], which combines ab initio gene predictions and homology evidence. SNAP v2006-07–28 [69], GeneMark.hmm-E v3.47 [70] and AUGUSTUS v3.3.3 [71] were used for *ab initio* gene prediction; SNAP and GeneMark were both trained on the UAMH 299 assembly, and *Fusarium graminearum* was used as the gene prediction species model for AUGUSTUS. CDS of Bb strain ARSEF 8028 [33] were supplied as expressed sequence tag (EST) evidence and the UniProtKB/Swiss-Prot database of protein sequences [72] was used as protein homology evidence. The gene predictions were processed using Genome Annotation Generator v2.0.1 [73], which added start and stop codons and removed transcripts with introns shorter than 10 bp or coding sequences shorter than 90 bp. Annotations that were missing a start and/or stop codon were then manually removed. The quality of the filtered annotations was assessed with GeneValidator v2.1.10 [74] using the TrEMBL database [72], and these scores were considered while selecting genes of interest in later analyses. Functional annotations were assigned to protein sequences by running stages 4 and 5 of the EnTAP [75] pipeline (v0.10.7-beta), which included a similarity search to the Uniref90 [72] database followed by GO term and Pfam domain assignment using InterProScan v5.30–69.0 [76]. Gene predictions that were not annotated by similarity search or gene family assignment were filtered out, and BUSCO [59] was run in protein mode to assess the completeness of the final gene annotations.

Orthogroups were inferred from the protein sequences using OrthoFinder v2.5.1 [34] with default parameters. The longest isoform of each gene was supplied for this analysis. OGs were functionally annotated with Gene Ontology terms and Pfam domains by selecting the most common terms and domains assigned to the genes included in each OG. Core orthogroups were identified as those that included one or more gene in every strain, and core, single-copy OGs were present in only one copy in each. Accessory OGs were identified as those present in two or more, but not all strains, and singleton OGs were those present only in one strain. Next, group-specific OGs were manually extracted by identifying those that included one or more genes from each strain of a given group, while not containing any genes from the strains in the other group. Functional relevance of orthogroup sets was assessed by performing GO enrichment analyses with clusterProfiler v3.18.1 [77]. Benjamini & Hochberg’s false discovery rate (FDR) [78] was used to correct the p-values for multiple comparisons, and significantly enriched GO terms were identified at alpha = 0.05.

### Biosynthetic gene cluster mining

The antiSMASH algorithm v5.1.2 [53] was used to identify biosynthetic gene clusters within the genomes using the –taxon fungi option. The MAKER annotations were supplied using the --genefinding-gff3 option. The runs included active site finder analysis (--asf), and clusters were compared against a database of antiSMASH-predicted clusters (--cb-general), known gene clusters from the MIBiG database (--cb-knownclusters) and known subclusters (--cb-subclusters). The fungal-specific Cluster Assignment by Islands of Sites (CASSIS) algorithm [79] was used to aid in the prediction of cluster regions by searching for conserved binding motifs in promoter regions using the --cassis option. The --cf-create-clusters option was used to find extra clusters, and Pfam and GO terms were mapped with --pfam2go.

### RNA Sample preparation for transcriptomic analysis

The starting fungal inoculum was reactivated from frozen mycelial stocks through spot inoculation at the centre of a PDA medium and incubated for 3–5 d at 25 °C. A one-cm^2^ agar block was cored on to the mycelial culture and transferred to four different pigment inducing media: CDAYE (for red pigmentation), Malt Extract Agar (MEA; for yellow pigmentation), Potato Dextrose Agar (PDA; for yellow pigmentation), and 0.25xSaboraud Dextrose Agar (SDA; for induction of conidiation). After comparative quality analysis of the cultures (i.e., morphology, pigmentation response, and conidiation density), the conidia from 0.25 × SDA were harvested, titered, and inoculated in 25 mL CDBYE broth medium in a 250 mL Erlenmeyer flask to a final concentration of 1.0 × 10^7^ conidia/mL and incubated for 72 h at 28 °C at 175 rpm. The active mycelial culture was inoculated (10% v/v inoculum) in a 100 mL CDBYE broth medium in a baffled 500 mL Erlenmeyer flask and incubated for 72–86 h at 28 °C at 175 rpm. The mycelial slurries were harvested using a Stericup Quick Release-GP vacuum filtration system (0.22 μm, polyethersulfone membrane; Millipore-Sigma, USA). The mycelial mat was aseptically transferred to a pre-weighed 50 mL conical tube, flash frozen in liquid nitrogen, and stored at -80 °C until processing. Quality control analysis was carried out, as described previously, for all the samples to determine bacterial contamination. The frozen mycelial mats were sent to the Michael Smith Laboratories, University of British Columbia, for RNA extraction.

### RNA Extraction

Three biological replicates for each of the 8 isolates, for a total of 24 samples, were extracted for total RNA. Starting with 2–5 g (with the exception UAX-29, which required 7–9 g), mycelium was ground with liquid nitrogen in a mortar and pestle to a fine powder. Kolosova et al.’s RNA extraction protocol [80] was used to process 100–200 mg of ground tissue. Instead of drying the RNA pellet for three minutes at room temperature, the sample was spun for an additional 30 s, remaining liquid was removed with a micropipette tip and samples were air-dried for one minute. The final pellet was resuspended in 30 µl of Nuclease-Free water. Total RNA concentration was determined using a NanoDrop 1000 (ThermoFisher Scientific) and assessed for quality on an Agilent 2100 Bioanalyzer and Agilent RNA 6000 Nano Kit LabChips. Total RNA (37.5 ng/µL) was sent to Canada’s Michael Smith Genome Sciences Centre for sequencing.

### Transcriptome sequencing

Qualities of total RNA samples were assessed using an Agilent Bioanalyzer RNA Nanochip and arrayed into a 96-well plate (ThermoFisher Scientific). Polyadenylated RNA was purified using the NEBNext Poly(A) mRNA Magnetic Isolation Module [E7490L] (New England Biolabs, Ipswich, MA, USA) from 1000 ng total RNA. Messenger RNA selection was performed using NEBNext Oligo d(T)25 beads (New England Biolabs) incubated at 65 °C for five minutes, followed by snap-chilling at 4 °C to denature RNA and facilitate binding of poly(A) mRNA to the beads. mRNA was eluted from the beads in NEBNext Tris Buffer from the NEBNext Poly(A) Magnetic Isolation Kit (New England Biolabs) and incubated at 80 °C for two minutes, then held at 25 °C for two minutes. RNA binding buffer was added to allow the mRNA to re-bind to the beads, mixed 10 times and incubated at room temperature for five minutes. The sample plate was placed on the magnet and the supernatant discarded. The mRNA bound beads were washed twice, then cleared again on magnet. The supernatant was again discarded, and mRNA was eluted from the beads in 20 µL Tris buffer incubated at 80 °C for two minutes. mRNA was transferred to a new 96-well plate.

First-strand cDNA was synthesized from heat-denatured, purified mRNA using a Maxima H Minus First Strand cDNA Synthesis kit (ThermoFisher Scientific) and random hexamer primers at a concentration of 200 ng/µL along with a final concentration of 40 ng/µL Actinomycin D, followed by PCR Clean DX (Aline Biosciences, Woburn, MA, USA) bead purification on a Microlab NIMBUS robot (Hamilton Company, Reno, NV, USA). The second strand cDNA was synthesized following the NEBNext Ultra Directional Second Strand cDNA Synthesis protocol (New England Biolabs) that incorporates dUTP in the dNTP mix, allowing the second strand to be digested using USER™ enzyme (New England Biolabs) in the post-adapter ligation reaction and thus achieving strand specificity.cDNA was fragmented by Covaris LE220 sonication to achieve 250–300 bp average fragment lengths. The paired-end sequencing library was prepared following the Canada’s Michael Smith Genome Sciences Centre strand-specific, plate-based library construction protocol on a Microlab NIMBUS robot (Hamilton Company). Briefly, the sheared cDNA was subject to end-repair and phosphorylation in a single reaction using an enzyme mix (New England Biolabs) containing T4 DNA polymerase, Klenow DNA Polymerase and T4 polynucleotide kinase, incubated at 20 °C for 30 min. Repaired cDNA was purified in 96-well format using PCR Clean dX beads (Aline Biosciences) and 3’ A-tailed (adenylation) using Klenow fragment (3’ to 5’ exo minus) and incubated at 37 °C for 30 min prior to enzyme heat inactivation. Illumina TruSeq adapters were ligated at 20 °C for 15 min. The adapter-ligated products were purified using PCR Clean DX beads, then digested with USER™ enzyme (1U/µL) (New England Biolabs) at 37 °C for 15 min followed immediately by 10 cycles of indexed PCR using NEBNext Ultra II Q5 Master Mix (New England Biolabs) and Illumina’s primer set. The PCR products were purified and size-selected twice using a 1:1 PCR Clean DX beads-to-sample ratio, and the eluted DNA quality was assessed with Caliper LabChip GX for DNA samples using the High Sensitivty Assay (PerkinElmer, Waltham, MA, USA) and quantified using a Quant-iT dsDNA High Sensitivity Assay Kit on a Qubit fluorimeter (Invitrogen) prior to library pooling and size-corrected final molar concentration calculation for Illumina HiSeq sequencing with paired-end 150 base reads.

The samples were submitted to the NCBI SRA under BioProject accession PRJNA877233. The corresponding BioSample accessions are presented in Table 5.

**Table 5:** BioSample accession information for *Beauveria bassiana* samples under investigation

Strain	Sample Title	Accession	Sample Name
UAMH 299	*Beauveria bassiana* strain UAMH 299	SAMN30686972	Bb_UAMH_299
UAMH 299-UVR	*Beauveria bassiana* strain UAMH 299 (UV-resistant derivative)	SAMN30686973	Bb_UAMH_299-UVR
UAMH 298	*Beauveria bassiana* strain UAMH 298	SAMN30686974	Bb_UAMH_298
UAMH 298-UVR	*Beauveria bassiana* strain UAMH 298 (UV-resistant derivative)	SAMN30686975	Bb_UAMH_298-YVR
UAMH 1076	*Beauveria bassiana* strain UAMH 1076	SAMN30686976	Bb_UAMH_1076
UAX-29	*Beauveria bassiana* strain UAX-29	SAMN30686977	Bb_UAX-29
UAMH 4510	*Beauveria bassiana* strain UAMH 4510	SAMN30686978	Bb_UAMH_4510
110.25	*Beauveria bassiana* strain 110.25	SAMN30686979	Bb_110.25

### Differential expression and Co-Expression analysis

Gene-level expression was quantified against CDS from the reference Bb strain ARSEF 2860 [32] with Salmon v1.5.2 [81]. Salmon was run in quasi-mapping mode and additionally corrected for sequence-specific and fragment-level GC biases using the --seqBias and --gcBias options. Differential expression analysis was carried out to using DESeq2 [82]. Gene expression was compared between the dark-red and pale-red strains, with the pale-red strains used as the reference level. Differential expression (DE) was tested using a log_2_ fold change (LFC) threshold of 1 and altHypothesis = ”greaterAbs” to identify upregulation and downregulation, and significantly DE genes were selected at FDR < 0.05. GO enrichment analysis was performed using clusterProfiler [77]. The ARSEF 2860 annotations were obtained for this analysis from AnnotationHubv2.22.1 [83] under the record AH86840, and significantly enriched terms were selected at FDR < 0.05. Genes with mean normalized count values of 0 were excluded from the background gene set for the GO enrichment analysis.

Differential co-expression was assessed using the DGCA R package (v1.0.2) [84]. Variance stabilizing transformation from DESeq2 was applied to the raw count data with blind = F, and genes in the lowest 25^th^ percentile of variant were filtered out. Co-expression was calculated between gene pairs consisting of all filtered genes and *OpS1* (BBA_08179) and *OpS3* (BBA_08181), and differential correlation was identified between dark-red and pale-red strains. *P*-values were adjusted using FDR and significantly co-expressed gene pairs were identified between dark-red and pale-red strains at FDR < 0.05.

## Supplementary Information

**Additional file 1. **Dataset S1

**Additional file 2. **Dataset S2

**Additional file 3. **Dataset S3

**Additional file 4. **Additional materials

## Data Availability

The sequencing data and assemblies generated for the current study are available in the NCBI SRA under BioProject accession PRJNA877233. The datasets generated and analyzed during the current study are included in this published article and its supplementary information files.

## Abbreviations

MPB: Mountain Pine Beetle
Bb: *Beauveria bassiana*
UV: Ultraviolet
BGC: Biosynthetic gene cluster
NRPS: Non-ribosomal peptide synthetase
PKS: Polyketide synthase
BUSCO: Benchmarking Universal Single-Copy Ortholog
OG: Orthogroup
GO: Gene Ontology
T1PKS: Type I polyketide synthase
CDS: Coding sequence
LFC: Log(2) fold change
TF: Transcription factor
ROS: Reactive oxygen species
CDBYE: Czapex-Dox Yeast Extract Broth
HPLC: High-performance liquid chromatography
CDAYE: Czapex-Dox Yeast Extract Agar
GEM: Gel Beads-in Emulsion
EST: Expressed sequence tag
CASSIS: Cluster Assignment by Islands of Sites
DE: Differential expression
