# Supplementary material for: Genomic virulence features of Beauveria bassiana as a biocontrol agent for the mountain pine beetle population
Source: BMC Genomics. 2023 Jul 10;24:390. doi: 10.1186/s12864-023-09473-4 (PMC10334655; doi:10.1186/s12864-023-09473-4)
Supplement: Supplementary file 4 — Additional file 4. Additional materials [file 12864_2023_9473_MOESM4_ESM.docx]

**Additional Materials**


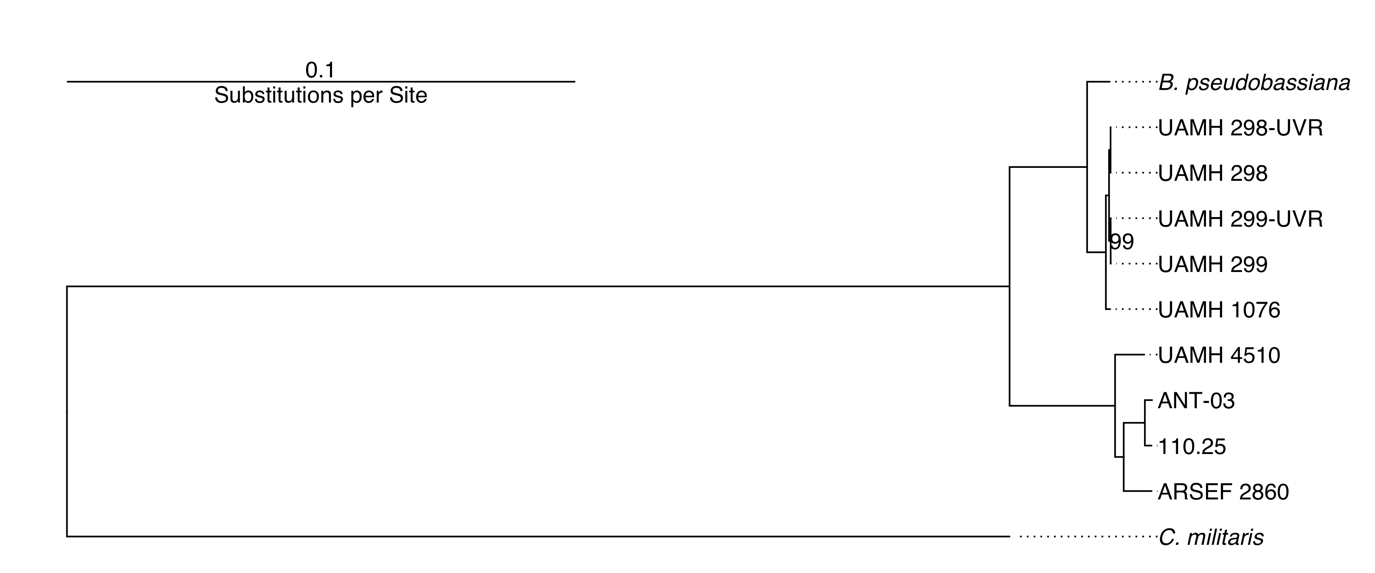


**Figure S1.** Complete BUSCO phylogeny of the eight *B. bassiana* strains under study.

The reference strain ARSEF 2860 and *Beauveria pseudobassiana* strain KACC 47484. *Cordyceps militaris* strain CM01 included as the outgroup. All non-labelled nodes have 100% bootstrap support.


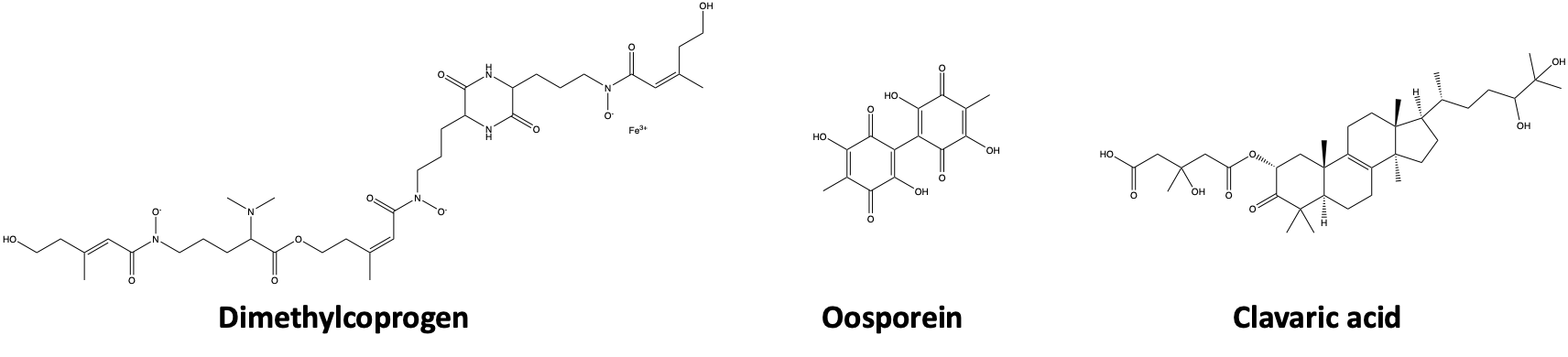


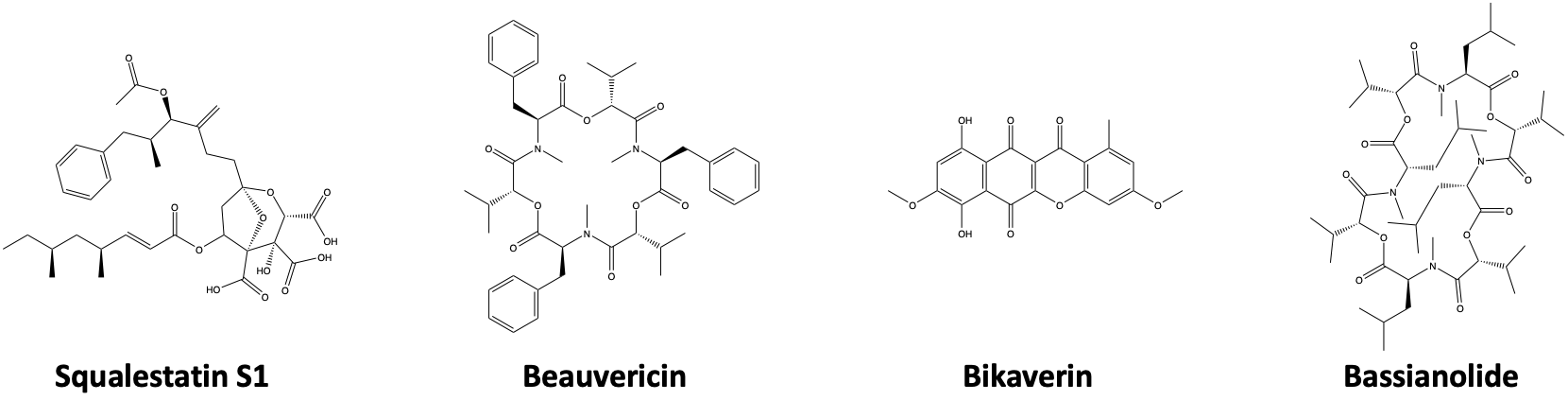


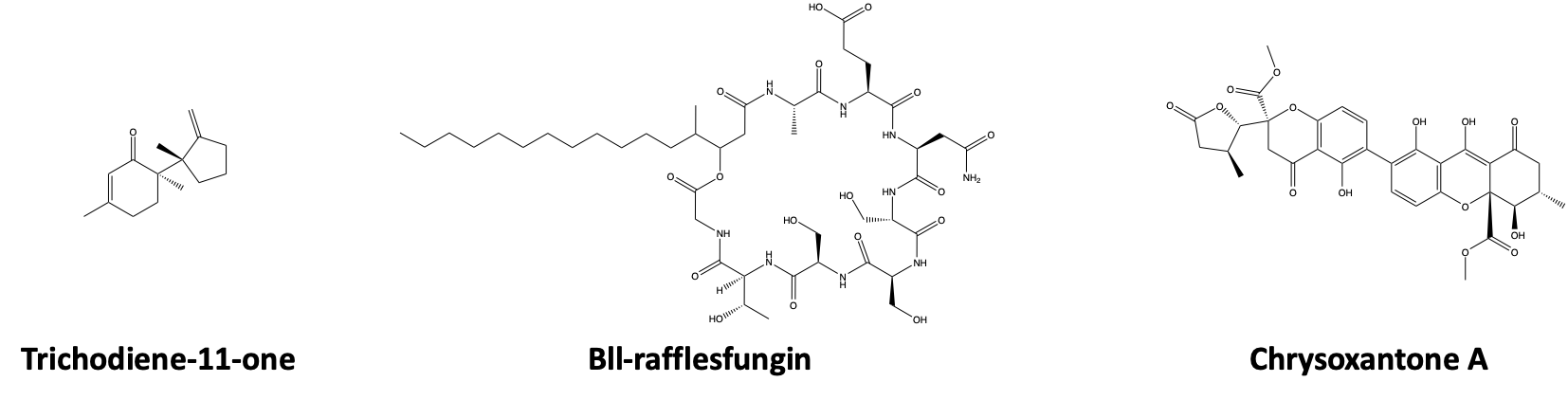


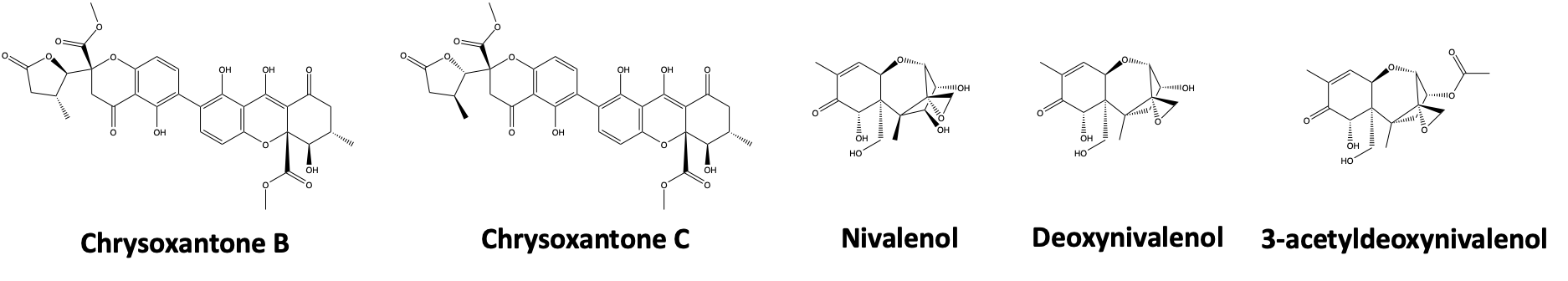


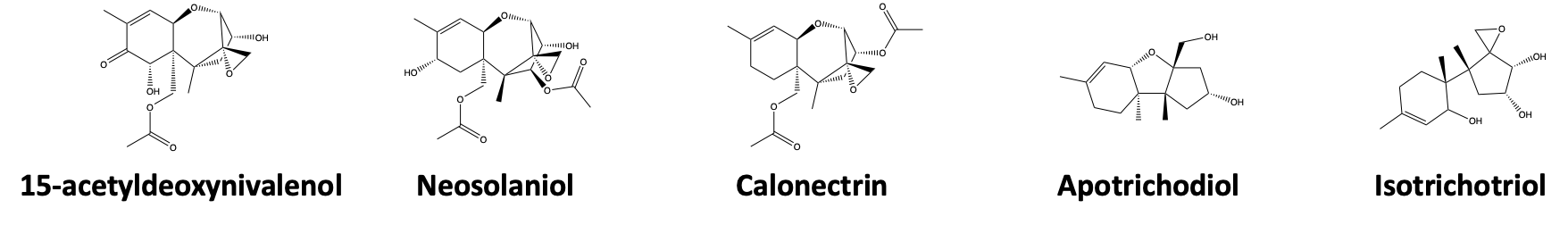


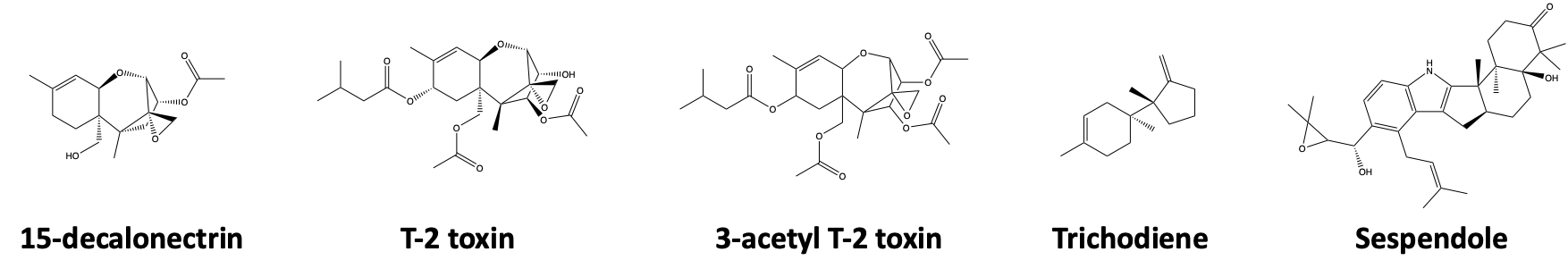


**Figure S2.** Chemical structures of common, group- and strain-specific gene clusters predicted by antiSMASH.
